# Effect of *Alocasia indica* Tuber Extract on Reducing Hepatotoxicity and Liver Apoptosis in Alcohol Intoxicated Rats

**DOI:** 10.1155/2014/349074

**Published:** 2014-05-29

**Authors:** Swagata Pal, Ankita Bhattacharjee, Sandip Mukherjee, Koushik Bhattacharya, Soumya Mukherjee, Suman Khowala

**Affiliations:** ^1^CSIR-Indian Institute of Chemical Biology, Drug Development and Biotechnology, 4 Raja S. C. Mullick Road, Kolkata 700032, India; ^2^Serampore College, Department of Physiology, Hooghly, West Bengal 712201, India; ^3^Drug Development Diagnostics and Biotechnology Division, CSIR-Indian Institute of Chemical Biology, Government of India, 4 Raja S. C. Mullick Road, Kolkata, West Bengal 700032, India

## Abstract

The possible protective role of ethanolic extract of *A. indica* tuber (EEAIT) in hepatotoxicity and apoptosis of liver caused by alcohol in rats was investigated. Treatment of rats with alcohol (3 g ethanol per kg body weight per day for 15 days intraperitoneally) produced marked elevation of liver biomarkers such as serum alanine aminotransferase (ALT), aspartate aminotransferase (AST), *γ*-glutamyl transpeptidase (*γ*-GT), and total bilirubin levels which were reduced by EEAIT in a dose-dependent manner. Furthermore, EEAIT improved antioxidant status (MDA, NO, and GSH) and preserved hepatic cell architecture. Simultaneous supplementation with EEAIT significantly restored hepatic catalase (CAT) and superoxide dismutase (SOD) activity levels towards normal. The studies with biochemical markers were strongly supported by the histopathological evaluation of the liver tissue. EEAIT also attenuated apoptosis and necrosis features of liver cell found in immunohistochemical evaluation. HPLC analysis of the extract showed the presence of three major peaks of which peak 2 (RT: 33.33 min) contains the highest area (%) and UV spectrum analysis identified it as flavonoids. It is therefore suggested that EEAIT can provide a definite protective effect against chronic hepatic injury caused by alcohol in rats, which may mainly be associated with its antioxidative effect.

## 1. Introduction

Alcoholic liver disease (ALD) is the most frequent cause of sustained excessive alcohol consumption [1]. ALD may take the forms of acute involvement (alcoholic hepatitis) or chronic liver disease (steatosis, steatohepatitis, fibrosis, and cirrhosis). Furthermore, sustained excessive alcohol intake favours the progression of other liver diseases, such as hepatotoxicity, which refers to liver dysfunction or liver damage [2].

Toxic substances generated during the metabolism of alcohol in the liver may contribute to the development of ALD. These substances include ROS that can destroy vital cell components through a process called oxidation. Recent studies have demonstrated that ROS and the resulting oxidative stress play a pivotal role in apoptosis. Antioxidants and overexpression of superoxide dismutase (MnSOD) can block or delay apoptosis of the vital cells [3]. Oxidative stress may also play a key role in apoptosis [4].

From the ancient past in India, several medicinal plants have been extensively used for the management of liver disorder.* Alocasia indica* is an indigenous herb belonging to family Araceae, traditionally used in inflammation and in diseases of abdomen and spleen [5]. This plant contains flavonoids, cyanogenic glycosides, ascorbic acid, gallic acid, malic acid, oxalic acid, alocasin, amino acids, succinic acid, and *β*-lectins [6]. The juice of leaves of the plant is used as digestive, laxative, diuretic, and astringent and for the treatment of rheumatic arthritis [7]. The leaves of* A. indica* showed antioxidant, antinociceptive, and anti-inflammatory activities and hepatoprotective activity against CCl_4_ induced liver damage model [8, 9]. It was reported that the leaves of the plant showed anthelmintic [10], antimicrobial [11], antidiarrheal, and* in vitro* antiprotozoal activities [12]. The rootstocks of the plant demonstrate free radical scavenging activity [13]. Alcoholic extracts of* A. indica* Schott. leaves (Araceae) showed antidiabetic and hypolipidemic effect in streptozotocin induced diabetic rats [14]. In a separate report, starch obtained from* A. indica *Linn. tubers (Araceae) was evaluated as a disintegrating agent [15]. In our previous study, the* in vitro* antioxidant activity and GCMS analysis of the ethanolic extract of* A. indica* tuber showed potential antioxidant activity with presence of bioactive phytosterol in the extract [16]. To the best of our knowledge, no other report was available using the tuber of the plant as hepatoprotectant against alcohol-induced liver damage. The objective of this study was to assess the hepatoprotective effects of ethanol extracted* A. indica* tuber extracts on the alcohol-induced liver damage rat model. This study also aimed to establish the correlation between antioxidative activity and antiapoptotic activity of the extract.

## 2. Methodology

### 2.1. Plant Material

The tuber vegetable (*Alocasia indica*) was collected from the local market of Kolkata, West Bengal, India, and was authenticated by the Botany Department of Serampore College, Hooghly, India. The tuber was chopped (2 × 2 inches) and dried under sun (40 ± 5°C) for a week. The dried tuber was finely powdered in grinder and sieved through the 40-micron sieve and stored in airtight containers.

### 2.2. Preparation of Ethanolic Extract of* A. indica* Tuber

100 g of the dried and powdered tuber of* A. indica* was extracted in 500 mL of 80% (v/v) ethanol for 72 h in Soxhlet apparatus, and the extract was centrifuged for 15 min at 4000 rpm. Supernatant was taken as ethanolic extract of* A. indica* tuber (EEAIT), concentrated using rotary evaporator at 40°C, dried in lyophilizer, and kept at −20°C for further use.

### 2.3. Induction of Experimental Hepatotoxicity by Alcohol

Female Wistar rats weighing 110 ± 4.5 g were kept in at the Central Animal House (IICB, Kolkata) at 12 h light/dark cycle and at 25 ± 2°C. All animal experiments were performed according to the ethical guidelines suggested by the Institutional Animal Ethics Committee (IAEC) of Indian Institute of Chemical Biology, Kolkata (IICB/AEC-APP/June meeting/2013). The animals were allocated into four groups with five rats in each group and provided with a control diet composed of carbohydrate (71%), protein (18%), fat (7%), and salt mixture (4%) [17]. The experimental group of animals received alcohol by intraperitoneal injection (i.p.) at the dose of 3 g ethanol (15%, v/v) per kg body weight per day for 15 days. Absolute ethanol was diluted with 0.9% (w/v) NaCl to get the desired concentration. EEAIT was also injected intraperitoneally by the following manner after performing the routine toxicity tests of the extract [9]. The experiment was designed as follows. 

*Group 1*. (C) Control animals received normal rat diet and water and were injected with normal saline (0.9% NaCl) for the whole experimental period.

*Group 2*. (E) Ethanol treated group had rats that received intraperitoneal injection of ethanol (3 gm/kg body weight/day) for 15 days. 

*Group 3*. (E + AI200) Low dose treated group had rats that received intraperitoneal injection of ethanol (3 gm/kg body weight/day) with EEAIT(200 mg/kg body weight/day) for 15 days.

*Group 4*. (E + AI400) High dose treated group had rats that received intraperitoneal injection of ethanol (3 gm/kg body weight/day) with EEAIT(400 mg/kg body weight/day) for 15 days.

*Group 5*. (S) Standard group had rats that received intraperitoneal injection of ethanol (3 gm/kg body weight/day) with silymarin (100 mg/kg body wt/day) for 15 days.

At the end of the experiment, all the rats were sacrificed by cervical dislocation. Blood serum was collected via cardiac puncture and subjected to serum biochemical analysis. Liver was immediately separated for biochemical, histopathological, and immunohistochemical evaluation. All experiments were performed in triplicate.

### 2.4. Serum Analysis

Activities of liver marker enzyme in blood serum, including AST, ALT, *γ*GT, and bilirubin, were measured using kits obtained from ACCUREX biomedical Pvt. Ltd.

### 2.5. Preparation of Liver Homogenate

Liver was cut into small pieces on ice and homogenized in 10% Tris HCl buffer (0.1 mole/L, pH 7.4) [17]. The homogenate was centrifuged at 10000 rpm at 4°C and the supernatant was collected for the estimation of NO, MDA, and SOD. For catalase and GSH estimation, isotonic phosphate buffer was used for homogenization of liver at pH 7.4, 0.1 M [17] and pH 8, 0.01 M [17], respectively, and then the tissue extracts were prepared according to the method mentioned above.

### 2.6. Estimation of Protein

Protein in the homogenate of liver tissue was estimated by the method of Lowry using BSA as standard [18].

### 2.7. Estimation of NO Production

NO production was estimated by Griess reaction, which was expressed in the form of nitrite accumulation. Liver homogenates (100 *μ*L) were loaded into microtitre plate followed by addition of 100 *μ*L Griess reagent (1% sulfanilamide in 5% H_3_PO_4_ and 0.1% naphthyl ethylene diamine dihydrochloride) and incubated at room temperature for 10 minutes. Later, the absorbance was taken at 550 nm using ELISA Reader (Thermo Scientific, USA) [17]. The results were expressed as *μ*mole/mg protein.

### 2.8. Estimation of Lipid Peroxidation

Lipid peroxidation was detected by measuring thiobarbituric acid reactive substance (TBARS). Two mL of liver homogenate was mixed with 1 mL of 20% (v/v) TCA and 1 mL of 0.67% (v/v) TBA and then boiled for 10 minutes. After cooling, the mixture was filtered through Whatman filter paper and the reading of filtrate was taken at 530 nm [17]. The amount (mmol) of MDA/mg protein was quantitated as an index of lipid peroxidation.

### 2.9. Estimation of GSH

In 200 *μ*L PBS, 20 *μ*L of liver extracts was mixed with 10 *μ*L dTNP (4 mg/mL in methanol) and then incubated at room temperature for 15 minutes. After that, readings were taken at 412 nm [17]. Results were expressed as mmol GSH/mg protein.

### 2.10. Estimation of Antioxidant Enzymes: SOD and CAT

Assay of SOD activity is based on the inhibition of NBT reduction by SOD. Briefly, 2.5 mL of 0.05 mol sodium carbonate buffer (pH 10) was mixed with 0.1 mL of 3 mmol/L EDTA, 3 mmol/L xanthine, 1.5 mg/mL bovine serum albumin, 0.75 mmol/L NBT, and the homogenates of liver. Reaction was initiated by adding 0.1 mL of 56 mU/mL xanthine oxidase [19]. After 30 min of incubation, the reaction was terminated by mixing 6 mmol/L CuCl_2_ and was centrifuged at 350 g for 10 min. Absorbance of blue formazan was recorded at 560 nm. The relative absorbance was then converted into unit of SOD activity per mg protein.

Catalase activity was determined by the decomposition of H_2_O_2_ at 240 nm at 25°C [20]. 0.2 mL of H_2_O_2_ solution (10 mmol/L dissolved in 50 mmol/L potassium phosphate buffer at pH 7.0) was mixed with 0.1 mL of liver homogenates and decrease of absorbance in every 30 s over a period of 3 min was recorded. Changes in the rate of absorbance were converted into unit of catalase/mg protein using a conversion factor (3.45), which corresponds to the decomposition of 3.45 micromoles of hydrogen peroxide in a reaction mixture producing a decrease in the absorbance from 0.45 to 0.40 units.

### 2.11. Histopathological Evaluation

After 15 days of treatment, livers of all animals were fixed in 10% formalin, embedded in paraffin, cut into 5-6 *μ*m by rotary microtome, and stained with haematoxylin-eosin to assess the histopathological changes.

### 2.12. TUNEL Assay

Liver sections were deparaffinized and rehydrated in descending alcohol concentrations. Apoptotic cells in liver were detected using Apo-BrdU-IHC* in situ* DNA fragmentation assay kit obtained from BioVision, USA.

### 2.13. Immunocytochemistry

Detection of NFkB and caspase-3 was done by the method of Giakoustidis et al., 2008 [21]. Deparaffinized and rehydrated liver sections were prepared for incubation with cleaved caspase-3 (Asp 175) antibody (Cell Signaling Technology Inc., Danvers, MA) at a dilution 1/200 or NF-kB p65 antibody at a dilution 1/1000 (Cell Signaling Technology Inc., Danvers, MA) overnight at 48°C. Sections were then incubated with extrAvidin peroxidase conjugates (Sigma-Aldrich) and finally were stained with DAB tablets (Sigma-Aldrich).

### 2.14. HPLC and UV Spectrum Analysis

HPLC analysis was conducted with a Shimadzu chromatograph equipped with photodiode array detector and a 4.6 × 250 mm reverse phase C18 column. Dried EEAIT was dissolved in appropriate 20% acetonitrile. The sample analysis of the sample was performed at room temperature, in the wavelength range of 254 at 1600 psi using a flow rate of 1.0 mL/min. The injection volume of samples was 50 *μ*L. Total run time was 60 minutes. The gradient elution of two solvents was used—solvent A (TFA 0.1% in water) and solvent B (TFA 0.1% in 100% acetonitrile). The gradient program was begun with 100% A and was held at this concentration for the first 5 minutes. This was followed by 100% eluent B for the subsequent 35 minutes after which the concentration of B was stable 100% for the next 5 minutes and then reduced to 0% in the next 10 minutes. UV spectrum analysis was done in parallel by scanning the chromatogram in a UV range of 200–400 nm in HPLC.

### 2.15. Statistics

Data were expressed as mean ± SE. Kruskal-Wallis nonparametric ANOVA test was performed to find whether or not scores of different groups differ significantly. To test intergroup significant difference, Mann-Whitney *U* multiple comparisons test was performed. Differences were considered significant if *P* < 0.05.

## 3. Results

### 3.1. Effect on Serums ALT, AST, *γ*GT, and Bilirubin Levels

Serums ALT and AST are two biochemical markers normally used for early stage assessment of liver injury. Alcohol-induced elevation of the two marker enzymes' serum ALT (76.63%, *P* < 0.01) and AST (52.63%, *P* < 0.01) levels was compared to normal group indicating the incident of liver injury (Table 1). Treatment with EEAIT at the low dose (200 mg/kg/day) displayed the recovery percentage of serums ALT (46.34%, *P* < 0.05) and AST (28.57%) followed by high dose (400 mg/kg/day) ALT (73.17%, *P* < 0.01) and AST (100% *P* < 0.01), when compared to alcohol treated group. Posttreatment with EEAIT recovered serum *γ*GT level by 42.39%, *P* < 0.05 and 75.72%, *P* < 0.01 at the concentration of 200 mg/kg/day and 400 mg/kg/day, respectively, which was increased drastically by 81.31%, *P* < 0.01 in alcohol toxicated group (Table 1). The same effect was observed in serum bilirubin (Table 1) level in which alcohol intoxication increased bilirubin level about four times (341.17%, *P* < 0.01) in comparison to the control group. EEAIT at the concentration of 200 mg/kg/day and 400 mg/kg/day could reduce the bilirubin level by 1.5 times (*P* < 0.01) and 2.8 times (*P* < 0.01), respectively, compared to alcohol treated group. The results are close to the results obtained by standard silymarin treated group with administration of EEAIT at the concentration of 400 mg/kg/day.

### 3.2. Effect on MDA, NO, and GSH Content in Liver Homogenate

MDA and NO levels were markedly increased in ethanol-attenuated liver, hallmarks of lipid peroxidation, and inflammatory response. Enhanced MDA (27.32%, *P* < 0.01) and NO (35.64%, *P* < 0.01) levels in alcohol treated rats were decreased by 46.19%, *P* < 0.01 and 70.72% in EEAIT treated rat at the concentration of 200 mg/kg/day (Table 1). The recovery percentage of EEAIT at 400 mg/kg/day was enhanced to 82.34%, *P* < 0.01 and 82.51% for MDA and NO, respectively.

In the alcohol treated group, there was a significant decrease in GSH content (23.57%, *P* < 0.01) as compared to control group. EEAIT at 200 mg/kg/day enhanced the GSH level by 17.27% (*P* < 0.05) but the result is statistically insignificant. The extract at concentration of 400 mg/kg/day can increase GSH level significantly (41.81%, *P* < 0.05) (Table 1). The results of liver marker enzymes, NO, MDA, and GSH, in the extract coadministered groups were comparable with that of the silymarin treated group.

### 3.3. Effect on the Level of SOD and CAT in Liver Homogenate

After being intoxicated with ethanol, a decline in the level of SOD (8.51%, *P* < 0.01) and catalase (67.12%, *P* < 0.01) was observed in liver injury groups (ethanol-induced) when compared to the normal group. SOD activity was back to normal by EEAIT at the lower concentration though catalase activity recovered significantly by 72.64% (*P* < 0.01) at the higher doses (Table 1).

### 3.4. Effect on Serum Levels of Insulin

**Figure 1:**
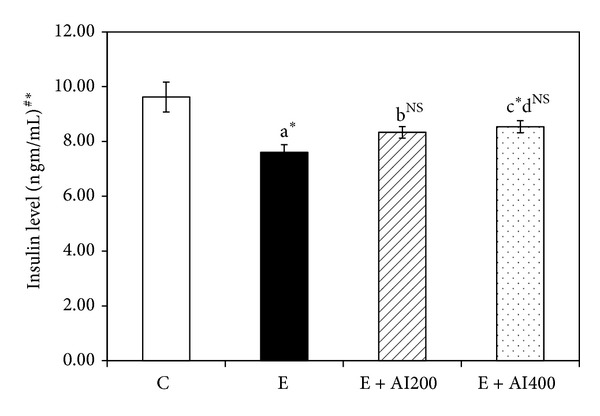
Effect of EEAIT (200 and 400 mg/kg body weight/day for 15 days i.p.) on insulin levels in all experimental groups. Values are expressed in mean ± SEM. Significance level based on ^#^Kruskal-Wallis nonparametric ANOVA and Mann Whitney *U* multiple comparison tests—^a^control versus ethanol, ^b^ethanol versus AI200, ^c^ethanol versus AI400, ethanol versus AI400, ^d^AI200 versus AI400. *P < 0.05, **P < 0.01, ***P < 0.001, N.S. not significant.

There was a significant decrease in the levels of insulin hormone in ethanol administration (*P* < 0.05). Supplementation with 400 mg/kg/day EEAIT significantly recovered ethanol-induced changes in plasma insulin level by 46.53% (*P* < 0.05) (Figure 1).

### 3.5. Histopathological Changes of Liver

**Figure 2:**
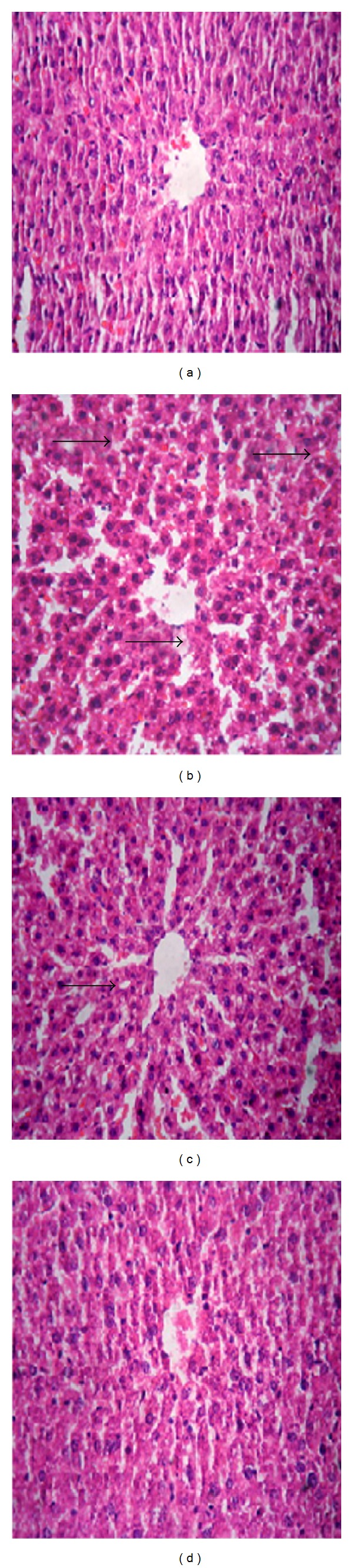
Photographs of liver histopathology. (a) Liver section from untreated or healthy control rats, exhibiting normal architecture. (b) Ethanol (3 gm/kg/day)—treated rats show the presence of fat droplets indicated by the black arrow. (c) and (d) Liver section from ethanol-intoxicated rats treated with EEAIT at 200 and 400 mg/kg/day, respectively, showed an absence of necrosis and regeneration of hepatocytes around the central vein. Hematoxylin and eosin (H&E) staining (40x).

Histological analysis (Figure 2) showed that there was no pathological abnormality observed in the liver of control rat (Figure 2(a)). In comparison to the normal liver architecture of the control group Figure 2(b) showed the alcohol-induced severe necrotic changes and other changes in liver section such as microvesicular steatosis, increase in sinusoidal space, inflammatory infiltration of lymphocytes, and dilation of the central vein with an increased number of fat droplets. These findings indicated early phases of liver injury in alcohol treated group. In the experimental groups, EEAIT at the concentration of 200 mg/kg/day (Figure 2(c)) showed the moderate protection in liver morphology as observed in the normal pattern of the central vein and radiating pattern of cell plates. On the other hand, EEAIT at the concentration of 400 mg/kg/day exhibited significant protection from liver damage (Figure 2(d)) as evidenced by noticeable recovery from ethanol-induced liver damage with fewer microvesicular steatoses, hepatocytes necrosis features, and absence of fat droplets.

### 3.6. TUNEL Assay of Treated and Untreated Liver Sections

**Figure 3:**
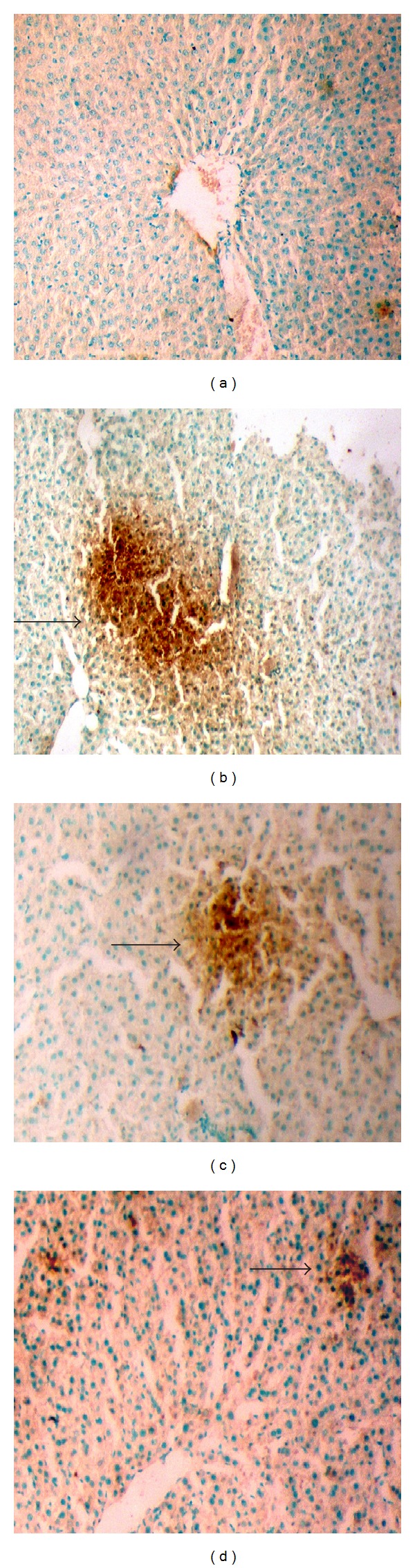
TUNEL assays of liver sections from rats treated with ethanol (3 gm/kg/day) and supplemented with EEAIT. Liver section from control (a), treated with ethanol (b), treated with ethanol and EEAIT at 200 mg/kg/day (c) and 400 mg/kg/day (d). Black arrow indicates apoptotic cells. Sections were taken at 40x magnifications.

The TUNEL assays (Figure 3) showed no apoptotic nuclei in the control liver tissue (Figure 3(a)), whereas in ethanol treated liver, large quantities of positive TUNEL cells were observed with numerous condensed and fragmented nuclei (brown in colour indicated by arrow, Figure 3(b)). In EEAIT treated group the quantities of positively stained cells were decreased significantly and simultaneously with increasing concentration of EEAIT from 200 to 400 mg/kg/day (Figures 3(c)-3(d)).

### 3.7. Immunocytochemistry

**Figure 4:**
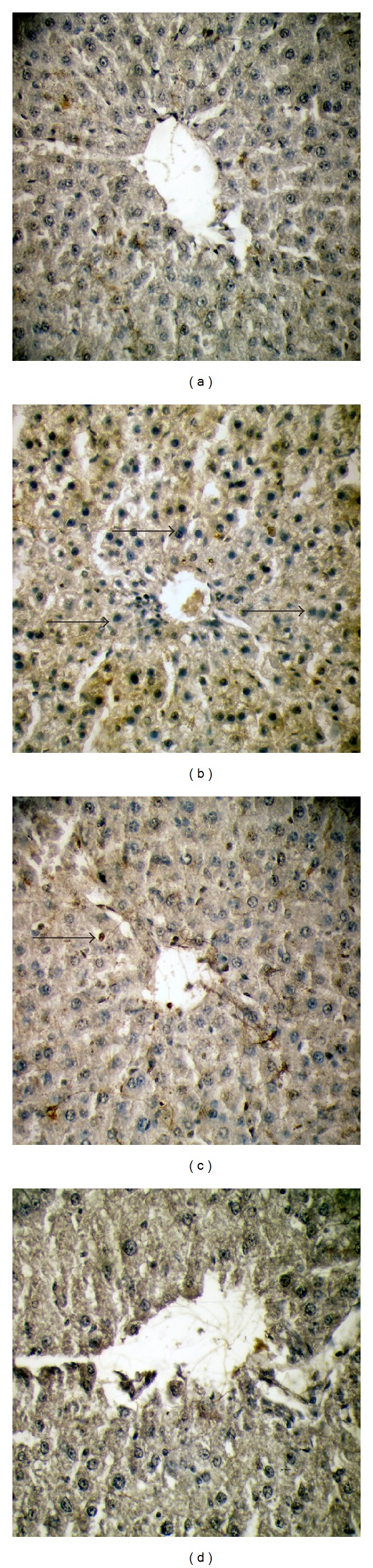
NF-kB p65 expression in immunohistochemistry of liver sections from rats in different experimental groups (a)–(d). (a) Liver section from control showed normal architecture of the liver section. (b) A huge amount of apoptotic dead cells was found in liver section of alcohol treated rat, which was indicated by the white arrow. (d) Moderate apoptotic bodies were shown by EEAIT administration at the concentration of 200 mg/kg/day. (d) Almost normal architect of the liver section found in EEAIT treatment at the concentration of 400 mg/kg/day. All the sections were taken at 40x magnifications.

**Figure 5:**
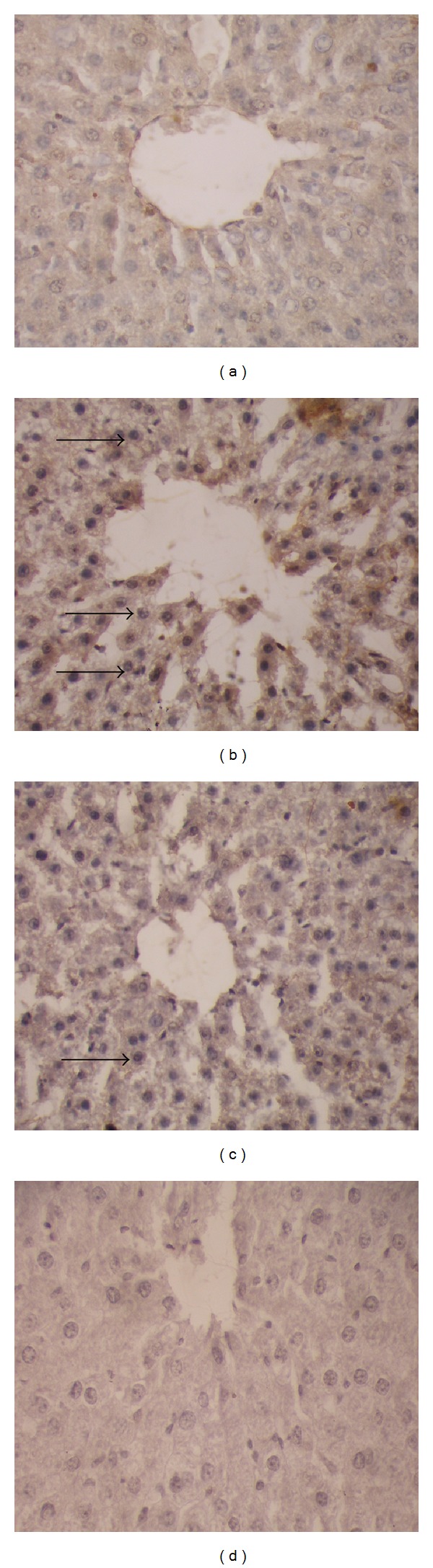
Expression of caspase-3 in immunohistochemistry. (a) Liver section from control showed normal architecture of the liver section. (b) A huge amount of apoptotic dead cell was found in alcohol treated liver indicated by the black arrow. (c) Moderate apoptotic bodies were shown by EEAIT administration at 200 mg/kg/day. (d) Almost normal architect of the liver section found in EEAIT treatment at the concentration of 400 mg/kg/day. All the sections were taken at 40x magnifications.

Activated NF-*κ*B was assessed by immunocytochemistry by using activation specific monoclonal antibodies to NF-*κ*B p65 subunit, the epitope of which binds only after I*κ*B dissociation. In this experiment, much fewer numbers of positively stained cells were observed in the control (Figure 4(a)) group, which was enhanced markedly in alcohol intoxicated liver sections (Figure 4(b)). Supplementation with EEAIT at the concentration of 200 mg/kg/day (Figure 4(c)) and 400 mg/kg/day (Figure 4(d)) can recover the changes as observed in simultaneous reduction of the positively stained cell number from lower dose to the higher dose. Similar patterns of results were also obtained in case of activated caspase-3 (Figures 5(a)–5(d)).

### 3.8. HPLC and UV Spectrum Analysis of EEAIT

**Figure 6:**
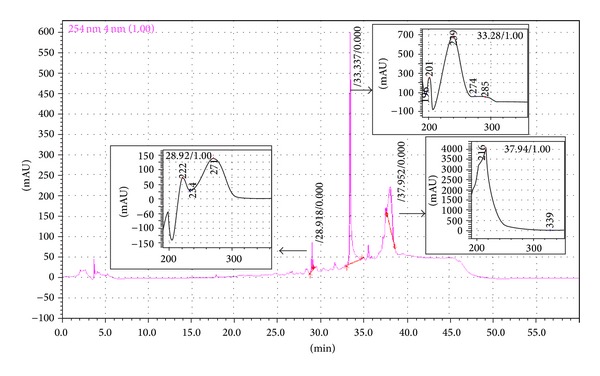
HPLC chromatogram of EEAIT and UV analysis of the three peaks RT: 28.91, 33.33, and 37.95 min (indicated by black arrow).

HPLC analysis of the crude EEAIT showed the presence of three major peaks at the retention time of 28.91 min, 33.33 min, and 37.95 min designated as peaks 1, 2, and 3, respectively, at 254 nm (Figure 6(a)). UV spectrum analysis of peak 1 showed maximum absorption at 222 nm and 217 nm, peak 2 at 201 and 239 nm, and peak 3 only at 216 nm.

## 4. Discussion

The present study showed the effect of the extract in reducing alcohol-induced changes in the liver of the rat model.

The hepatoprotective effects of EEAIT were assessed by measuring the serum markers such as AST, ALT, GGT, and bilirubin in an* in vivo *study in alcohol intoxicated rat. Excessive alcohol administration can accumulate NADH, which is the obvious fate of alcohol metabolism by alcohol dehydrogenases. Enhanced NADH production can synthesize more fatty acids and triglycerides and the leakage of cellular enzymes into plasma associated with serum ALT and AST [22]. Moreover, excess alcohol consumption has been linked with altered liver metabolism with leakage of cytoplasmic liver enzyme *γ*GT into blood [23]. Markers of severe alcoholic hepatitis or cirrhosis also include elevated levels of bilirubin into the plasma. Besides, results from histological images that showed accumulations of fatty droplets in the hepatocytes also provided clear evidence that the preinduction with alcohol (3 gm/kg/day) produced liver damage, including loss of cell membrane integrity and accumulation of fatty acids, in the rat. Thus, by restoring the level of serums ALT, AST, GGT, and bilirubin back to normal in alcohol intoxicated rat, a high dose (400 mg/kg/day) of EEAIT has certified its hepatoprotective effects against alcohol intoxication at least in part by reducing fat droplets in liver. Phenolic compound present in EEAIT [16] may also give membrane stability and repair liver damage caused by alcohol [24]. Liver is the main target organ of insulin and decrease in insulin level by alcohol can provide the dysfunction features of the pancreas which also indirectly affects liver functions. EEAIT also recovered the hormone level which is drastically decreased by alcohol.

GSH depletion is considered to be the chief factor leading to lipid peroxidation [25]. Earlier report revealed that alcohol was capable of generating by ROS glutathione synthesis inhibitor, producing glutathione loss from the tissue, and increasing MDA levels [26]. In our experiment increased amount of MDA in alcohol-induced liver signifies the enhanced degree of lipid peroxidation, which can lead to liver damage. Supplementation of EEAIT (200 and 400 mg/kg/day) decreased GSH level with a concomitant increase in MDA level as expected.

NO is an inflammatory mediator and highly reactive oxidant produced by iNOS, released by kupffer cells upon exposure to hepatotoxins such as alcohol [27]. Overproduction of NO by chronic alcohol exposure may stimulate the synthesis of some cytokines, such as interleukin-1 (IL-1), IL-2, IL-6, IL-8, and TNF-*α*, which in turn stimulate the synthesis of NO [28]. In our study increased production of NO in the ethanol group was restored to the normal level by EEAIT.

As a consequence of the constant oxidative challenge, cells have used their antioxidant systems (SOD and catalase) to counter the peroxidant fluxes [29]. A decrease in both activities in liver tissue of the alcohol-induced group was largely due to the impairment of antioxidant enzymes, which can protect the cells against ROS. Higher amount of total phenolic content and strong antioxidant activity in EEAIT [16] may enhance both the enzyme activities.

A plausible justification for hepatoprotective and antioxidant effects of EEAIT may be due to the presence of flavonoids and phenolic acids, which were highly detected particularly in ethanolic extraction method [16]. In the present study, histological evaluation was undertaken to support the biochemistry profiles. Elevation of the level of liver function biomarkers ALT, AST, *γ*GT, and bilirubin in blood was detected along with the decrease in antioxidant enzyme activity and severe necrosis in histopathological changes by 15-day administration of alcohol at the dose of 3 gm/kg/day. Liver injury hallmarks such as inflammation, necrosis, and fat droplets were restored back close to normal after administration of high dose (400 mg/kg/day) of EEAIT. The result was supported by the decrease in ALT, AST, *γ*GT, bilirubin, NO, and MDA and increase in GSH level, CAT, and SOD activities. Our observation also proves that EEAIT restores the liver injury in a dose-dependent manner.

Indication of apoptosis by TUNEL assay is morphologically characterized by some cellular changes, including DNA fragmentation and the appearance of cytoplasmic apoptotic bodies [30]. In our study, the cell death was detected by an increase in the apoptotic cell number in chronic alcohol-exposed rat livers by TUNEL assay, which was recovered by administration of EEAIT.

Recent investigations suggest that the induction of NF-*κ*B-dependent gene expression in Kupffer cells contribute to alcohol-induced liver injury [31]. In this study downregulation of NF-*κ*B signalling by EEAIT in ethanol-induced injury was due to suppression of NF-*κ*B-dependent target genes in alcohol treated liver. It was also reported that caspase-3 activation in the ethanol treated liver occurred mostly in the hepatocytes around the central vein [32]. Increased expression of caspase-3 was observed in alcohol-induced rat in TUNEL assay and consequence reduction of the expression in EEAIT treatment supports the antiapoptotic action of the extract. The finding was supported by other reports [33]. So the result suggests that activation of NF-*κ*B and caspase-3 may contribute to hepatocyte death in alcohol treated rats. Supplementation with 200 mg/kg/day and 400 mg/kg/dayEEAIT in ethanol treated rats successfully reduced these effects of alcohol in liver.

Qualitative analysis EEAIT in reverse phase HPLC showed three consecutive peaks at different retention time of which peak 1 (RT: 28.91 min) and peak 2 (RT: 33.33 min) showed a typical UV absorption spectrum pattern of flavonoid compounds [34] whereas peak 3 (37.95 min) showed maximum absorption in 217 nm wavelength, which may be another secondary compound and have to be analysed. MS analysis and other studies of the three compounds are in our future project.

## 5. Conclusions

The results strongly imply the potential use of EEAIT in future application for treatment in reducing oxidative stress, alcoholic liver disease, and apoptotic cell death of vital organs such as liver, which can create a lot of possibilities in therapeutic aspects.

## Figures and Tables

**Table 1:** Effect of EEAIT on the biochemical parameters in ethanol-induced hepatotoxicity in rats.

Parameter	C	E	E + AI200	E + AI400	E + silymarin	Kruskal-Wallis nonparametric ANOVA
ALT (U/L)	21.4 ± 3.4	37.8 ± 4.5^a∗∗^	30.2 ± 2.6^b∗^	25.8 ± 2.4^c∗∗d∗^	24.2 ± 1.7**	*P* < 0.01
AST (U/L)	26.6 ± 3.8	40.6 ± 3.4^a∗∗^	36.4 ± 4.4^b∗^	26.7 ± 6.1^c∗∗d∗^	26.6 ± 4.4**	*P* < 0.01
*γ*GT (U/L)	7.6 ± 1.0	13.78 ± 2.3^a∗∗^	11.16 ± 2.2^b∗^	9.1 ± 0.9^c∗∗d∗^	8.5 ± 1.4**	*P* < 0.01
Bilirubin (U/L)	0.17 ± 0.04	0.75 ± 0.1^a∗∗^	0.49 ± 0.05^b∗∗^	0.26 ± 0.06^c∗∗d∗∗^	0.19 ± 0.02**	*P* < 0.001
NO (µM/mg protein)	58.6 ± 7.0	79.4 ± 7.7^a∗∗^	64.6 ± 2.1^b∗∗^	62.2 ± 3.7^c∗∗d∗^	60.3 ± 2.3**	*P* < 0.01
GSH (mM/mg protein)	9.3 ± 0.86	7.13 ± 0.46^a∗∗^	7.5 ± 0.65^bNS^	8.05 ± 0.56^c∗d∗^	9.1 ± 0.8**	*P* < 0.01
MDA (mM/mg protein)	77.9 ± 2.8	99.3 ± 0.88^a∗∗^	89.4 ± 3.1^b∗∗^	81.7 ± 1.5^c∗∗d∗∗^	78.7 ± 1.3**	*P* < 0.001
SOD (U/mg protein)	0.3 ± 0.004	0.31 ± 0.002^a∗∗^	0.34 ± 0.006^b∗∗^	0.35 ± 0.004^c∗∗d∗∗^	0.34 ± 0.001**	*P* < 0.001
CAT (U/mg protein)	153.8 ± 11.4	50.6 ± 28.1^a∗∗^	69.4 ± 36.4^bNS^	125.6 ± 17.8^c∗∗d∗∗^	149.7 ± 12.3**	*P* < 0.01

Significance level based on Kruskal-Wallis nonparametric ANOVA and Mann Whitney *U* multiple comparisons tests—^a^control versus ethanol, ^b^ethanol versus AI200, ^c^ethanol versus AI400, ethanol versus AI400, and ^d^AI200 versus AI400. *P < 0.05, **P < 0.01, ***P < 0.001, NS: not significant (mean ± SD).

## Abbreviations

EEAIT: Ethanolic extract of* A. indica* tuber
ALD: Alcoholic liver disease
ROS: Reactive oxygen species
SOD: Superoxide dismutase
i.p.: Intraperitoneal injection
NaCl: Sodium chloride
AST: Aspartate aminotransferase
ALT: Alanine aminotransferase
*γ*GT: Glutamyl transpeptidase
HCl: Hydrochloric acid
NO: Nitric oxide
MDA: Malonaldehyde
SOD: Superoxide dismutase
GSH: Glutathione
BSA: Bovine serum albumin
H_3_PO_4_: Phosphoric acid
TBARS: Thiobarbituric acid reactive substance
TCA: Trichloroacetic acid
TBA: Thiobarbituric acid
PBS: Phosphate buffer saline
dTNP: 2,2′-Dithiobis 5 nitropyridine
NBT: Nitroblue tetrazolium
EDTA: Ethylenediamine tetraacetic acid
CuCl_2_: Cupric chloride
H_2_O_2_: Hydrogen peroxide.
